# Advances in the study of OSA and diabetic foot

**DOI:** 10.1186/s13098-022-00842-9

**Published:** 2022-05-12

**Authors:** Jiayu Lin, Hailing Song, Meihong Liang, Zeqiang Cai, Tan Chen, Zhenyu Lin, Jinying Zhang

**Affiliations:** 1 Department of Endocrinology, The Second Affiliated Hospital of Fujian Medical University, Quanzhou, 362000 China; 2 Fujian Medical University, Fuzhou, 350100 China; 3 Department of Neurology, The Second Affiliated Hospital of Fujian Medical University, Quanzhou, 362000 Fujian China

**Keywords:** Diabetic complications, Diabetic foot, Obstructive sleep apnea, Pathogenesis

## Abstract

Diabetic foot is one of the most serious and painful chronic complications of diabetic patients, especially elderly diabetic patients. It has a high rate of death, disability and amputation. Obstructive sleep apnea (OSA) is a treatable chronic sleep disorder. Existing evidence suggests that OSA may promote the development and delay the healing of diabetic foot, and continuous positive airway pressure therapy may promote the healing of ulcers. Therefore, in the multidisciplinary diagnosis and treatment of diabetes, cooperation with sleep medicine should be strengthened, and the basic and clinical research on diabetic foot combined with OSA should be strengthened, so as to reduce the amputation rate, improve the cure rate and reduce the incidence of cardiovascular events.

## Introduction

Diabetic foot (DF) is one of the most serious and painful chronic complications of patients with diabetes, especially elderly patients with diabetes. It has a high rate of death, disability and amputation, which seriously affects the quality of life of patients and brings great burden to patients, families and society [1,2]. The annual incidence of diabetic foot ulcer in China is about 8.1%, and the 1-year recurrence rate of foot ulcer after healing is about 31.1% [1]. Obstructive sleep apnea (OSA) is the most common chronic sleep disorder with a prevalence of 4–24% in the population [3], and its prevalence is increasing with obesity and age. The pathophysiological mechanism of OSA is the repeated occurrence of complete or incomplete upper airway obstruction during sleep, accompanied by intermittent hypoxemia, hypercapnia and sleep architecture disorders. It is an important risk factor for type 2 diabetes, hypertension, cerebrovascular disease, cardiovascular diseases and other disorders [4,5]. In addition, limited data shows that the prevalence of OSA in patients with chronic trauma is as high as 57%, which exceeds the prevalence of OSA in the general middle-aged population [6], suggesting that OSA may contribute to the development of skin ulcers and even delay the healing of ulcers, leading to the development of chronic ulcers. Since OSA can lead to the development of diabetes and the incidence of OSA is significantly higher in diabetic patients [7], what is the prevalence of OSA in diabetic foot patients? Does it lead to the development of foot ulcers? Does it aggravate the severity of foot ulcers and delay their healing? Given the complexity of OSA, the relationship between OSA and DF is far than the above and deserves deeper consideration.

## Obstructive sleep apnea and diabetes

### Bidirectional relationship between obstructive sleep apnea and diabetes

Cross-sectional studies have shown that the prevalence of type 2 diabetes in patients with OSA ranges from 15 to 30%, and the prevalence of elevated glucose and insulin resistance is significantly higher in OSA patients than in the healthy population [8], with a dose-dependent risk. The co-prevalence of OSA in patients with type 2 diabetes was found to be about 71%. Intermittent hypoxia and sleep fragmentation in patients with OSA have been found to be associated with dysglycemia, insulin resistance, and abnormal islet β-cell function. To date, there have been prospective studies looking at the prevalence of type 2 diabetes in OSA patients with a follow-up period of 3 to 16 years, with inconsistent results across studies after correction for confounders. Another cross-sectional study found that diabetic patients with OSA had worse glycemic control than those without OSA [9]. Currently, about more than half of all diabetic patients worldwide may be affected by both diabetes and OSA. The possible consequence is that patients with combined OSA may not have effective glycemic control. Based on current clinical findings, we have observed a higher prevalence of diabetes in patients with OSA [10]. However, it is not yet possible to conclude whether OSA will lead to the development of type 2 diabetes over time. Future studies should focus on the relationship between OSA and diabetes using strict criteria and longer follow-up in larger populations. The relationship between OSA and diabetes should also be addressed.

A follow-up study [11] with an average follow-up of 67 months showed that the initial severity of OSA and its physiological consequences could predict the risk of subsequent diabetes in OSA patients, controlling for multiple confounding factors. The current study demonstrates a strong association between OSA and insulin resistance, glucose intolerance and the risk of type 2 diabetes. And OSA is independently associated with poor glycemic control. A study of the association between OSA and diabetes showed that OSA patients have a higher BMI than patients with other conditions, and OSA is associated with endocrine diseases, especially diabetes [12, 13].

### Two-way mechanism between obstructive sleep apnea and diabetes

Results of prospective cross-sectional studies in indicating an independent association between OSA severity and insulin resistance in patients without type 2 diabetes, and short-term lab-based studies in healthy human subjects have demonstrated that sleep restriction, sleep fragmentation and intermittent hypoxemia can lead to glucose metabolism disorders [14].

In OSA, increased energy expenditure due to the increase of resting metabolic rate may induce compensatory neuroendocrine adaptation to increase hunger and food intake beyond energy balance requirements, leading to excess energy and a higher risk of obesity, leading to dyslipidemia, inflammatory status, and lower insulin sensitivity [15]. The specific pathophysiological mechanisms are shown below:

1. Increased sympathetic activity: Patients with OSA have increased sympathetic nerve activity, and intermittent hypoxemia and sleep structural disorders during sleep further stimulate sympathetic nerves leading to greater blood glucose fluctuations and insulin resistance [16], while hyperinsulinemia causes excessive stimulation of the carotid body, leading to increased sympathetic adrenal activity and blood flow, forming a vicious circle and exacerbating abnormal glucose metabolism [17].
2. Intermittent hypoxia: Intermittent hypoxemia affects insulin sensitivity and impairs β-cell function, causing increased hepatic glycogen output and pancreatic oxygenation stress, which can increase fasting glucose levels by 67% and decrease glucose tolerance by 27%; even after correction of hypoxia, impaired glucose tolerance, insulin resistance and pancreatic β-cell function persist [18].
3. Hypothalamic–pituitary–adrenal (HPA) axis dysfunction: Increased sympathetic nerve activity in OSA patients can activate the hypothalamic–pituitary–adrenal axis, resulting in increased glucocorticoid secretion. Glucocorticoid can promote glucose synthesis and glycogen decomposition, leading to insulin resistance.
4. Systemic inflammatory response syndrome: This includes the release of pro-inflammatory mediators such as TNF-α and interleukin-6. The researchers also found elevated circulating levels of C-reactive protein, reactive oxygen species and advanced glycation end products in OSA patients [19].
5. Changes in adipocytokines, such as increased leptin levels and decreased adiponectin levels.
6.  Fatigue and lethargy caused by OSA can lead to decreased body activity and increase the risk of diabetes, which is another mechanism of OSA causing diabetes. Sleep deprivation can lead to insulin resistance.

Diabetic autonomic dysfunction is a risk factor for OSA [20]. Diabetic patients with peripheral neuropathy, especially autonomic nerve dysfunction, have an increased risk of sleep and respiratory disorders [21]. Chronic hyperglycemia leads to structural damage and functional impairment of the divine meridian through oxidative stress, which leads to functional impairment of the divine meridian through impaired control function of the central axis of respiration, leading to sleep and sleep respiratory disorders [22]. On the other hand, diabetic autonomic neuropathy may aggravate the collapse of the upper respiratory tract, reduce the diameter of the upper respiratory tract, affect the reaction of the upper respiratory tract due to the destruction of the laryngeal dilator muscle, and increase the susceptibility to obstructing respiratory suspension sleep disorders induced by OSA [23] or painful peripheral neuropathy [22].

## OSA and diabetic foot

The two pathophysiological mechanisms of diabetic foot are diabetic peripheral neuropathy (DPN) and lower extremity arterial diseases (LEAD). Diabetic polyneuropathy (DPN) is a common chronic complication of diabetes. It is characterized by loss of sensation in the distal lower extremities, while severe neuropathic pain may also be present. DPN is the cardinal risk factor of diabetic foot ulceration mediated by unrecognized acute or chronic trauma-mediated diabetic foot ulcers [24]. OSA and nocturnal hypoxia were significantly associated with peripheral neuropathy in nondiabetic patients, independent of age and obesity. A study examining the relationship between OSA and DPN found that type 2 diabetes patients with OSA were four times more likely to develop DPN than patients without OSA. The severity of DPN was independently correlated with the degree of nocturnal hypoxia in OSA, and the lower the minimum oxygen saturation, the higher the prevalence of DPN [25]. The recurrent hypoxic process resembles ischemia-reperfusion injury. It can produce large amounts of reactive oxygen species (ROS) in a variety of ways. The ROS produced by intimal oxidative stress can directly damage the protein, nucleic acid and lipid of nerve tissue and interfere with the respiratory chain of mitochondria, resulting in the damage of nerve structure and function [26]. A small study also found that OSA was associated with axonal degeneration. Continuous positive airway pressure (CPAP) is one of the most effective non-surgical treatments for OSA. CPAP can reduce sleep apnea, alleviate daytime sleepiness, and improve the patient’s prognosis. Six months of continuous positive airway pressure (CPAP) can improved neural action potential amplitude [27]. In type 2 diabetes, Patients with severe OSA were 3 times more likely than those without or with mild OSA to develop a chronic complication associated with diabetes [28]. Results of a meta-analysis showed that compared with diabetic patients without DPN, The OR value of OSA in diabetic patients with DPN was 1.95 [29]. Later, Gu and others [30] found that OSA was significantly correlated with DPN. Further studies have shown that diabetic patients with OSA may increase oxidative stress and damage microcirculation [25] to promote the activation of PARP and reduce the density of nerve fibers in the epidermis [31], thus leading to the occurrence of neuropathy.

Intermittent hypoxemia will not only increase reactive oxygen species and oxygen free radicals, activate different cascades, positively regulate inflammatory reactions, cause endothelial cell secretion dysfunction, promote cell apoptosis, destroy vascular endothelial integrity, lead to endothelial dysfunction, and then lead to extensive vascular damage [32], but also promote the excessive proliferation of vascular smooth muscle cells, This leads to the occurrence and development of atherosclerosis [33]. OSA promotes the occurrence and severity of diabetic LEAD. A study shows that the prevalence of LEAD in OSA patients diagnosed by polysomnography (polysomnography is currently the gold standard for diagnosing OSA) is about 98%, and LEAD grading increases with the severity of OSA. Age and apnea hypopnea index (AHI) are important risk factors for arterial plaque [34]. In addition, in patients with moderate and severe OSA, the atherosclerotic plaque was larger than that in the control group [35]. In OSA patients treated with continuous positive airway pressure (CPAP), arterial stiffness can be improved [36, 37].

In patients with poor sleep quality, the number of immune cells in the skin is lower than those with good sleep quality [38], and the wound healing is also delayed [39]. In patients with diabetes mellitus complicated with OSA, because of intermittent hypoxemia and sleep disorder, is it possible that the number of skin immune cells is low, resulting in the occurrence of wound and refractory wounds? Studies have shown that in patients with diabetes mellitus complicated with OSA, due to prolonged inflammation, oxidative stress, sympathetic activation, physiological adaptation to hypoxia, reactive oxygen species in reactive oxygen species, reactive oxygen species act as the second messenger in the cell, play a role in regulating the biochemical functions of vascular smooth muscle cells and the chemical pathway of fibroblast proliferation, and so on. Reactive oxygen species can cause different degrees of endothelial cell damage, and then affect its normal physiological function [32]. And decreased mobilization of endothelial progenitor cells (ERC) and EPC apoptosis, resulting in the reduction of circulating EPCs, resulting in the reduction of endothelial cell repair ability, which leads to the delay of wound healing [36].

OSA is closely related to the onset of diabetic foot [31]. OSA may lead to the occurrence of diabetic foot through the following mechanisms:

1. OSA promotes the occurrence and development of diabetic foot by inducing DPN.
2. OSA promotes diabetic foot by inducing or aggravating LEAD damage.
3. OSA leads to a decrease in circulating EPCs, which leads to a decrease in endothelial cell repair and promotes the occurrence of diabetic foot.
4. OSA patients are often accompanied by obesity, which increases plantar pressure and increases the risk of foot ulcer [24].

## OSA and prognosis of diabetic foot

In diabetic patients, the prevalence of diabetic foot was much lower in patients without OSA than in patients with varying degrees of OSA. This suggests that patients with both diabetes and OSA should be aware of the risk of diabetic foot. Although an earlier study by Andrews et al. [40] did not find that OSA could impair the healing of some DF amputation wounds (79.2% of diabetic patients), the study was a retrospective, observational study and the reliability of the findings is debatable. Subsequently in an analysis based on case reports, it was observed that undiagnosed severe OSA may impede the rate of healing of diabetic foot ulcers. Continuous positive airway pressure (CPAP) is one of the treatments for OSA. The effects of CPAP therapy on overall glycemic control remain contradictory, it may be that CPAP is more effective in patients with poorer glycemic control at baseline. However, it is important to entertain other potential benefits of CPAP [41]. In the absence of peripheral arterial disease or infection, severe OSA may delayed healing of the foot ulcers while CPAP treatment may promote healing of the ulcers. Recently, Maltese used S T O P-B A N G score [27] to judge OSA, score ≥ 4 to diagnose OSA. Follow-up 12 months showed that the healing rate of diabetic foot ulcer patients with score ≥ 4 was lower than that of diabetic foot ulcer patients with score < 4. This suggests that in diabetic foot patients, the OSA delayed ulcer healing. In addition, Vas et al. [42] described three patients with type 2 diabetes and obesity who investigated how severe OSA interfered with healing of the diabetic foot. DFU healing was significantly improved in patients treated with OSA under continuous positive airway pressure, while no improvement was observed in patients who refused OSA treatment. Despite the small number of cases, the results are promising and may represent a breakthrough and good prognosis for DF treatment with similar characteristics. Therefore, the presence and severity of OSA should be considered in the treatment and prevention of DF. Physicians should consider OSA as one of the major risk factors for the development of diabetic foot. In addition, DF specialists and sleep disorder specialists can work in a multidisciplinary manner with patients with OSA and DF to address prevention and treatment strategies.

There were some limitations in this study. With the exception of CPAP, this study did not elucidate whether weight loss through lifestyle interventions, pharmacotherapy, or weight loss interventions have been shown to be effective in reducing OSA severity in obese patients with type 2 diabetes. This study focused on the association between OSA and type 2 diabetes. However, the prevalence of OSA is significantly higher in type 1 diabetic patients than in the general population. The epidemiology of OSA in patients with type 1 diabetes, its relationship with the diabetic foot, and the effects of CPAP treatment remain to be studied. Future studies should explore the accurate evaluation of the effects of OSA treatment on glucose metabolism and diabetic complications in prediabetes, type 1 diabetes, and type 2 diabetes.

## Conclusions

In summary, OSA is closely linked to the patient’s endocrine disease, which can lead to the development of diabetes and diabetic foot, and the presence of diabetes can aggravate the symptoms of sleep apnea, OSA is correlated with diabetic foot. The available evidence suggests that OSA can promote diabetic foot development and delay wound healing, while CPAP treatment may promote ulcer healing. Therefore, for patients with refractory diabetic foot ulcer, it is significant for clinicians to diagnose whether patients have OSA. Positive continuous positive airway pressure (CPAP) should be a promising adjunct to the treatment of diabetic foot with OSA. Therefore, we should not ignore the sleep science in the diagnosis and treatment of diabetic foot. We should strengthen the cooperation with the sleep medicine specialty and strengthen the basic and clinical research of diabetic foot combined with OSA, which will help to reduce the amputation rate and improve the cure rate, reduce the incidence of cardiovascular events.

## Data Availability

Not applicable.
